# Outcomes of Nissen sleeve gastrectomy in a short‑term follow‑up: a new future?

**DOI:** 10.20452/wiitm.2025.17945

**Published:** 2025-04-17

**Authors:** Paweł Lech, Dominika Mysiorska, Julia Młyńska, Emilia Szymborska, Natalia Dowgiałło‑Gornowicz

**Affiliations:** Department of General, Minimally Invasive and Elderly Surgery, School of Medicine, University of Warmia and Mazury in Olsztyn, Olsztyn, Poland

**Keywords:** bariatric surgery, gastroesophageal reflux disease, gastroesophageal reflux disease remission, Nissen sleeve gastrectomy, reflux

## Abstract

**INTRODUCTION:**

Sleeve gastrectomy (SG) is the most commonly performed bariatric procedure worldwide, offering significant weight loss and remission of obesity-related diseases. However, SG is associated with a high risk of gastroesophageal reflux disease (GERD). To mitigate this issue, a modified procedure combining SG with Nissen fundoplication (Nissen-sleeve gastractomy [NSG]) has been developed.

**AIM:**

The primary aim of this study was to assess the effectiveness of NSG in GERD remission. Secondary objectives included the evaluation of short-term weight loss and the resolution of obesity-related diseases.

**MATERIALS AND METHODS:**

We conducted s prospective analysis of 34 patients who underwent NSG between September 2023 and August 2024 at a single center in Poland. Inclusion criteria comprised age over 18 years, eligibility for metabolic and bariatric surgery, and GERD symptoms. Data on GERD severity and weight loss outcomes were collected before and after the surgery.

**RESULTS:**

Mean (SD) preoperative body mass index was 40.3 (4.4) kg/m². GERD symptom severity decreased from a median score of 8 to 1 (*P* <⁠0.001), with 97.1% of the patients achieving GERD remission. Mean (SD) percentage of total weight loss was 23% (6.3%) at 6 months and 30.3% (6.7%) at 12 months postoperatively. Type 2 diabetes remission was observed in 28.6% of the patients, and hypertension remission, in 58.3%. No postoperative complications were reported.

**CONCLUSIONS:**

NSG is a safe and effective procedure that leads to GERD remission, while achieving short-term satisfactory weight loss and mitigating obesity-related diseases.

## INTRODUCTION

Sleeve gastrectomy (SG) has become the most widely performed bariatric procedure worldwide, showing the fastest growth rate over the past decade.^1^**^,^**^2^ It has surpassed Roux-en-Y gastric bypass (RYGB) due to shorter procedure time and less anatomical alteration, while achieving similar outcomes in weight loss and remission of obesity-related diseases.^3^ However, despite its increasing popularity and clinical success, there are concerns regarding its potential association with gastroesophageal reflux disease (GERD).^4^

For patients with obesity and severe GERD, RYGB has traditionally been the preferred bariatric procedure.^5^**^,^**^6^ SG alters the esophagogastric angle, weakens the lower esophageal sphincter by dividing the muscular sling fibers, reduces gastric volume, and increases intragastric pressure, all of which contribute to a higher likelihood of postoperative GERD and hiatal hernia.^7^**^,^**^8^

Given the benefits of SG and the risk of GERD, a modification of the standard SG technique has been developed. In 2016, Nocca et al^9^ proposed a combined procedure of SG and Nissen fundoplication (Nissen sleeve gastrectomy [NSG]). This combined approach addresses GERD while preserving the advantages of SG in terms of weight loss and remission of obesity-related comorbidities. NSG results in sustained weight loss, significant improvement in GERD, and a low reoperation rate over a 5-year follow-up period.^10^

## AIM

The primary aim of this study was to report the early outcomes of NSG surgery in terms of GERD remission in the department where this procedure was first introduced. The secondary objectives included evaluating short-term weight loss and the resolution of associated comorbidities.

**TABLE 1:** Characteristics of the study population (n = 34)

Parameter		Value
Age, y, mean (SD)		39.8 (10.3)
Sex, n	Men	3
	Women	31
Preoperative BMI, mean (SD)		40.3 (4.4)
T2D, n (%)		7 (20.6)
Hypertension, n (%)		12 (35.3)
PPIs, n (%)		34 (100)
Gastroscopy findings, n (%)	Hiatal hernia	27 (79.4)
	Los Angeles classification of esophagitis, grade A	13 (38.2)
	Los Angeles classification of esophagitis, grade B	9 (26.5)
Severity of symptoms, median (IQR)		8 (7–9)
Chronic cough, n (%)		21 (61.8)

Abbreviations: BMI, body mass index; IQR, interquartile range; PPIs, proton pump inhibitors; T2D, type 2 diabetes

**TABLE 2:** Outcomes of Nissen sleeve gastrectomy

Parameter	Value	Min–Max
Operative time, min	60 (10.4)	45–85
T2D remission, n (%)	2 (28.6)	–
Hypertension remission, n (%)	7 (58.3)	–
Cough remission, n (%)	17 (81)	–
1 month after surgeryª		
BMI, kg/m²	36.8 (4.3)	29.3–45.5
%EWL	24.5 (9.4)	5–47.6
%TWL	8.8 (3)	14.2–36.2
6 months after surgery		
BMI	31 (4.2)	25.3–41.5
%EWL	63.4 (17.9)	29.3–98
%TWL	23 (6.3)	14.2–46.2
Severity of symptoms, median (IQR)	1 (1–1)	1–7
12 months after surgeryᵇ		
BMI, kg/m²	27.5 (3)	24.1–32.7
%EWL	84.6 (18.2)	51.6–107
%TWL	30.3 (6.7)	20–40
Severity of symptoms, median (IQR)	1 (1–1)	1–7

Data are presented as mean (SD) unless indicated otherwise.

a Data available for 34 patients

b Data available for 9 patients

Abbreviations: %EWL, percentage of excess weight loss; NS, Nissen sleeve gastrectomy; %TWL, percentage of total weight loss; others, see TABLE 1

## MATERIALS AND METHODS

This was a prospective analysis of patients undergoing laparoscopic NS surgery at a single center in Poland between September 2023 and August 2024. In order to be included in the study, the patients had to be over 18 years, meet the eligibility criteria for metabolic and bariatric surgery, present GERD symptoms before the surgery, and have GERD history with at least a 6-month follow-up. Patients with missing or inconsistent data were excluded from the study.

The following GERD-related data were collected: preoperative gastroscopy findings (hiatal hernia based on the Los Angeles classification of esophagitis), GERD impact scale (IS) scores before the surgery and 6 months postoperatively, GERD severity assessed using a Likert scale from 1 to 10 (1 indicating no symptoms and 10 representing the most severe ones), daily use of proton pump inhibitors (PPIs), and chronic cough.^11^ Additionally, we collected demographic data, such as sex, age, preoperative body mass index (BMI), presence of obesity-related diseases (type 2 diabetes [T2D] and hypertension), complications, and weight loss outcomes expressed as the percentage of excess weight loss (%EWL) and the percentage of total weight loss (%TWL).^12^ The data were collected during in-person patient visits.

### Surgical techniques and perioperative care

All patients underwent NSG according to the technique proposed by Nocca et al.^13^ The procedure began with the dissection of the esophageal hiatus and mobilization of approximately 6 cm of the esophagus from the mediastinum to the peritoneum. All patients underwent primary suturing of the diaphragmatic crura, both posteriorly and anteriorly to the esophagus, using a continuous barbed suture. The suturing was calibrated with a 36-F nasogastric tube.

The fundus of the stomach was completely released, as in SG. A 360-degree fundoplication wrap was then created from the fundus of the stomach around the distal esophagus and secured with 3 single sutures, with the middle one fixed to the esophagus. Next, SG was performed using a 36-F tube. Stapling was carried out while continuously moving the calibration tube. The last 2 staple firings were made approximately 1 cm from the wrap to ensure full blood supply. No hernia mesh was used in any of the patients. The wrap and the esophagus were not fixed to the diaphragm or the crura.

### Statistical analysis

All data were analyzed using Statistica 13.PL software (StatSoft Inc., Tulsa, Oklahoma, United States). A descriptive statistical analysis was conducted. The normality of distribution was assessed using the Shapiro–Wilk test. Categorical variables were presented as counts with percentages, and numerical variables, by means with standard deviations (SD), or medians with interquartile ranges (IQR), as appropriate. The Wilcoxon signed-rank test was used to compare preoperative and postoperative GERD-IS scores, as the data were paired. *P* values below or equal to 0.05 were considered significant.

#### Ethics

All data were anonymized. The study was conducted in accordance with the ethical standards of the 1964 Declaration of Helsinki and its subsequent amendments. The study was approved by the Bioethics Committee of the University of Warmia and Mazury in Olsztyn (23/2024). All patients provided written informed consent to participate in the study.

**TABLE 3:** Comparison of gastroesophageal reflux disease impact scale before and after Nissen sleeve gastrectomy

Questions	Before NSG, median (IQR)	After NSG, median (IQR)	*P* value
How often have you had pain in your chest or behind the breastbone?	2 (1–3)	4 (3–4)	0.004
How often have you had a burning sensation in your chest or behind the breastbone?	2 (1–3)	4 (3–4)	<0.001
How often have you had regurgitation or an acidic taste in your mouth?	2 (1–3)	4 (3–4)	<0.001
How often have you had pain or a burning sensation in your upper stomach?	3 (2–4)	4 (4–4)	<0.001
How often have you had a sore throat or hoarseness related to your heartburn or acid reflux?	3 (2–4)	4 (4–4)	<0.001
How often have your symptoms prevented you from getting a restful sleep?	3 (2–4)	4 (4–4)	0.004
How often have your symptoms prevented you from eating or drinking any of the foods you like?	2 (2–3)	4 (3–4)	0.001
How often have your symptoms prevented you from being fully productive at work or during daily activities?	2 (1–3)	4 (4–4)	0.003
How often have you taken additional medicines other than doctor-prescribed medication (eg, Maalox, Alusal, Manti)?	3 (1–4)	4 (4–4)	<0.001

Where: 1 – daily; 2 – often; 3 – sometimes; 4 – never

Abbreviations: see TABLE 1

## RESULTS

### Patients

A total of 36 patients underwent NSG at least 6 months before the analysis. Two patients were lost to follow-up, resulting in the follow-up rate of 94.4%.

The final analysis included 34 patients (31 women [91.2%] and 3 men [8.8%]). Mean (SD) preoperative BMI was 40.3 (4.4) kg/m² (TABLE 1). The majority of the patients (79.4%) had a hiatal hernia before surgery, and most (61.8%) suffered from chronic cough. According to patient-reported outcomes, median (IQR) severity of GERD before surgery was 8 (7–9) on a 1–10 Likert scale.

### Outcomes

Mean (SD) operative time was 60 (10.4) minutes. Mean (SD) %TWL after 6 months was 23% (3%), and after 12 months, 30.3% (6.7%; TABLE 2 ).

A total of 28.6% of the patients achieved T2D remission, while 58.3% experienced hypertension remission during the follow-up period. Additionally, 81% of the patients reported cough remission. Median (IQR) severity of GERD symptoms was 1 (1–7) on the Likert scale. Only 1 patient (2.9%) reported persistent GERD symptoms after NSG. There were no postoperative complications.

### Gastroesophageal reflux disease impact scale questionnaire

All patients completed the GERD-IS questionnaire. An improvement was observed in the answers provided to all 9 questions of the GERD-IS (TABLE 3).

## DISCUSSION

Our study reports the results 6 months after NSG in a high-volume bariatric center in Poland. These are the first patients operated on at this center using this procedure. To our knowledge, this is the first report on NSG in Poland.

Since its introduction into metabolic and bariatric surgery, SG has gained increasing popularity and is now the most commonly performed procedure due to its relatively high safety profile, technically straightforward approach, and excellent metabolic outcomes, as compared with other bariatric procedures.^1^**^,^**^3^ However, it has its drawbacks and complications. While the overall risk of early and late complications remains low, there is growing concern regarding the high incidence of postoperative reflux-related issues. Recent studies report that the incidence of GERD following SG can reach up to 52% after 12 months.^4^**^,^**^14^ Reflux complications now appear to be among the leading causes of revision surgery after SG.^15^

Although RYGB is considered a good option for patients with obesity and pre-existing reflux, due to its specific complications, it is not suitable for all patients.^16^ Limitations include the inability to perform comprehensive endoscopic surveillance postoperatively, a higher risk of complications in patients with inflammatory bowel disease, and potential concerns in women planning pregnancy, as the procedure is associated with a risk of internal hernias.^17^**^,^**^18^

NSG appears to be effective in reducing GERD symptoms. A total of 97.1% of the patients who underwent NS reported complete remission of symptoms at 6-month follow-up. The SM-BOSS (Swiss Multicenter Bypass or Sleeve Study)^19^ reported that the de novo GERD rate after SG may reach 31.6%. Olmi et al^20^ conducted a randomized study comparing NSG and SG. They included patients without GERD symptoms before surgery. According to their findings, 1 year after surgery, PPIs were necessary in 4.3% of the NS patients, as compared with 17.1% of the SG patients (*P* = 0.001). NSG may also have a protective effect against de novo GERD, which remains a significant concern after SG.

All patients completed the GERD-IS questionnaire before surgery and 6 months postoperatively. The analysis of GERD-IS demonstrated improvement in all 9 questionnaire domains. Notably, subjective relief of reflux symptoms was reported by all patients within a few days postoperatively. Particularly significant was the resolution of chronic cough and hoarseness. After 6 months, 17 patients (81%) no longer experienced these symptoms, a highly satisfactory outcome, in comparison with purely antireflux procedures.^21^

Hypertension remission was observed in 7 out of 12 patients (58.3%), while T2D remission occurred in 2 out of 7 participants (28.6%). These results are consistent with those reported in studies on other procedures.^22^**^,^**^23^ %EWL in our study was similar to that observed in randomized controlled trials comparing NSG and SG after a short follow-up.^20^ However, NSG could be expected to result in slightly lower weight loss than SG, due to the preservation of a larger portion of the gastric fundus. Long-term results of NSG described by Nocca et al^10^ are similar to those reported by Salminen et al.^16^

There are initial reports of early complications of NSG.^24^^,^^25^^,^^26^ However, in our study, no early or late complications were observed during a 6-month follow-up. The length of hospital stay was not prolonged, and no new postoperative symptoms were reported. The creation of the fundoplication wrap did not lead to additional postoperative discomfort or complications. A lack thereof is likely related to refinements in the originally described technique.

The patients who underwent NSG in our department were selected if they presented GERD symptoms. Based on our own observations, ideal candidates for this procedure were young patients with GERD symptoms, a hiatal hernia, and a BMI between 35 and 45 kg/m^2^. Smoking was a contraindication due to the risk of wrap necrosis and impaired tissue healing. Any inflammatory changes detected during preoperative endoscopy had to be fully resolved before surgery.

With the continuous evolution of metabolic and bariatric surgery, the development of new techniques is inevitable. Increasing evidence suggests that SG has a pro-reflux effect, leading to the worsening of pre-existing symptoms and the de novo emergence of GERD. Introducing a procedure similar to SG which would incorporate reflux protection, appears to be a logical step. NSG maintains similar weight loss results and obesity-related disease outcomes, while offering superior antireflux benefits. As compared with RYGB, NSG preserves the natural gastrointestinal anatomy and allows for complete endoscopic surveillance. Surgical techniques continue to evolve, and an increasing number of studies with large patient cohorts and long-term follow-up are being conducted^10^. Moreover, there is a clear need to standardize the NSG technique.

NSG can only be performed as a primary bariatric procedure in patients who have not undergone prior bariatric surgery. It is not suitable as a revisional procedure. However, its significant advantage is the potential for wrap reversal, allowing for a conversion back to a standard SG if necessary, or in the event of procedure-specific complications.

### Limitations

The main limitations of our study included its single-center design, same surgical team analyzing patient data, a relatively small sample size, and a short follow-up period, all due to the novelty of the procedure. A larger proportion of patients with metabolic complications of obesity, such as hypertension, T2D, and sleep apnea, would help better assess the long-term resolution of these conditions. Additionally, the lack of randomization in patient selection was a limitation, as only patients with GERD symptoms were included, introducing potential bias. Another limitation of the study was the lack of control endoscopy to evaluate GERD resolution. Further studies with larger patient cohorts are necessary, and such research is currently planned at our center. After analyzing a significantly larger group of patients, we anticipate satisfactory long-term outcomes, as the NSG procedure appears promising. This is supported by the findings of Savvala et al^10^ who reported favorable results in a 5-year follow-up, particularly regarding reflux symptoms relief.

## CONCLUSIONS

NSG is a safe procedure that, in the short-term, leads to GERD remission. Additionally, it yields satisfactory outcomes in terms of weight loss and improvement in obesity-related diseases.
